# Supplementary material for: Precision Beekeeping Systems: State of the Art, Pros and Cons, and Their Application as Tools for Advancing the Beekeeping Sector
Source: Animals (Basel). 2023 Dec 24;14(1):70. doi: 10.3390/ani14010070 (PMC10778344; doi:10.3390/ani14010070)
Supplement: Supplementary file 1 [file animals-14-00070-s001.zip › animals-2752536-supplementary.pdf]

Supplementary Materials

Table S1. Sensors' type equipped on the survey's considered PBS.

ID System	Weight	Internal Temperature	External Temperature	Wet	Humidity	Sound	Traffic Counting	Weather Station	Theft/GPS	Cam	Light
1	✓							✓	✓		
2	✓	✓			✓	✓			✓		
3	✓	✓		✓			✓	✓	✓	✓	
4	✓	✓			✓		✓ **	✓			
5	✓	✓			✓	✓			✓		✓
6		✓			✓	✓					
7	✓	✓			✓		✓	✓			
8	✓	✓			✓	✓					
9	✓								✓		
10	✓	✓		✓		✓					
11	✓	✓			✓				✓		
12	✓	✓			✓	✓		✓	✓		
13	✓	✓	✓		✓				✓		
14	✓	✓	✓		✓	✓					
15	✓ *	✓			✓	✓		✓			
16	✓	✓			✓	✓					
17	✓	✓			✓			✓	✓		
18	✓	✓	✓		✓	✓			✓		
19	✓	✓			✓		✓				
20	✓	✓	✓		✓	✓					
21	✓	✓			✓	✓			✓		
22		✓	✓		✓	✓					
23		✓	✓		✓	✓		✓	✓		
24	✓	✓	✓		✓		✓				
25	✓	✓	✓	✓	✓			✓	✓		
26	✓	✓		✓	✓			✓			

27	✓	✓	✓	✓	✓				✓		
28	✓	✓			✓						
29	✓										
30	✓	✓			✓						
31									✓		
32		✓			✓	✓					
Total	27	28	9	5	26	15	5	10	13	1	1

* Multiple intra-frame sensors; ** it can discriminate number of incoming and outgoing bees.

Table S2. Commercial precision beekeeping systems found in the web search.

PB System/Firm	Website	Country
BeeGuard	www.beeguard.it	ITA
3Bee	www.3bee.com	ITA
Melixa S.r.l.	www.melixa.it	ITA
Bee Hive Monitoring	www.beehivemonitoring.com	SVK
ApisProtect	www.apisprotect.com	IRL
arnia	www.arnia.co	GBR
BuzzBox	www.osbeehives.com	USA
HiveGenie	www.hivegenie.com	USA
Forsage	www.forsage.net	CZE
Antifurtoarnia	www.antifurtoarnia.it	ITA
Beenalytics	www.beenalytics.com	RUS
Arniaperfetta	www.arniaperfetta.it	ITA
Beehold	-	SRB
Sms scale	www.smsvaga.com	SRB
Beep	www.beep.nl	NLD
Bee and me	-	AUT
Solution bee	www.solutionbee.com	USA
Iobee	www.io-bee.eu	FRA
Gobuzzr	www.gobuzzr.com	IND

Beemate	www.beemate.buzz	AUS
Beesage	www.beesage.co	NLD
Intelligent hives	www.intelligenthives.eu	POL
Hyper hyve	www.hyperhyve.com	USA
Beewise	www.beewise.ag	ISR
HiveGenie	https://www.hivegenie.com	USA
Xlogbee	www.xlogbeescale.com	HRV
wolfwaagen	www.wolf-waagen.de	DEU
Savebees	www.save-bees.com	GRC
Optibee	www.optibee.fr	FRA
Opnehivescale	www.openhivescale.org	FRA
Beewatch	www.beewatch.de	DEU
nectar	www.nectar.buzz	CAN
Apisprotect	https://apisprotect.com	IRL

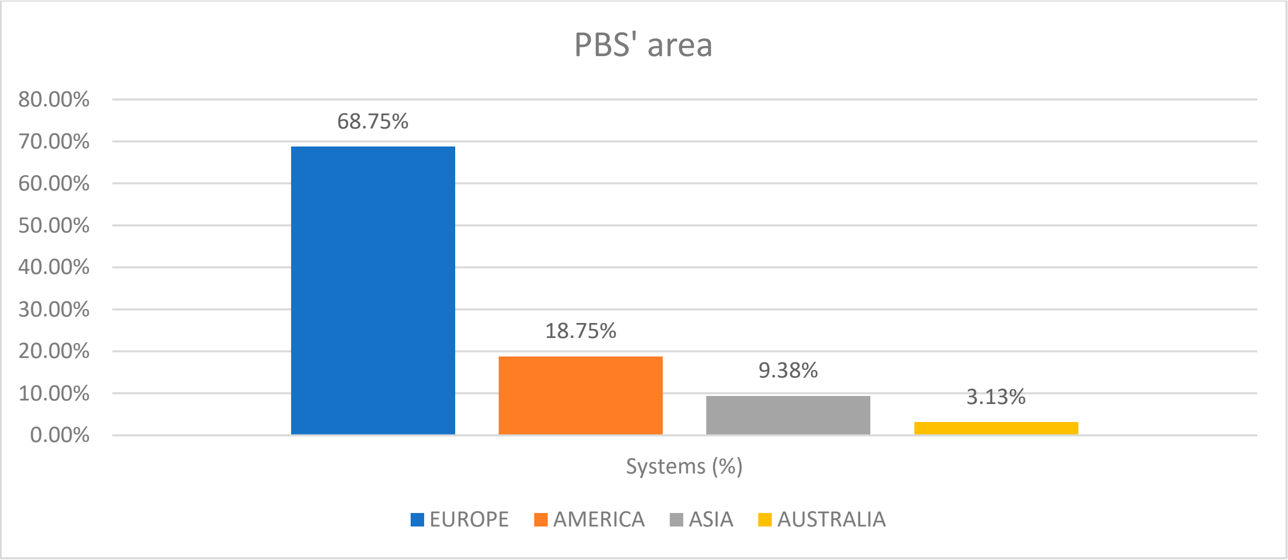

Figure S1. Percentage of systems analyzed in the survey in different areas of the world.
